# Genomic analysis of dominance effects on milk production and conformation traits in Fleckvieh cattle

**DOI:** 10.1186/1297-9686-46-40

**Published:** 2014-06-24

**Authors:** Johann Ertl, Andrés Legarra, Zulma G Vitezica, Luis Varona, Christian Edel, Reiner Emmerling, Kay-Uwe Götz

**Affiliations:** 1 Institute of Animal Breeding, Bavarian State Research Centre for Agriculture, Prof.-Dürrwaechter-Platz 1, Poing-Grub 85586, Germany; 2 INRA, UMR 1388 Génétique, Physiologie et Systèmes d’Elevage, CS 52627, Castanet-Tolosan 31326, France; 3 Université de Toulouse INPT ENSAT, UMR 1388 Génétique, Physiologie et Systèmes d’Elevage, Castanet-Tolosan 31326, France; 4 Departamento de Anatomía, Embriología y Genetíca, Universidad de Zaragoza, Zaragoza 50013, Spain

## Abstract

**Background:**

Estimates of dominance variance in dairy cattle based on pedigree data vary considerably across traits and amount to up to 50% of the total genetic variance for conformation traits and up to 43% for milk production traits. Using bovine SNP (single nucleotide polymorphism) genotypes, dominance variance can be estimated both at the marker level and at the animal level using genomic dominance effect relationship matrices. Yield deviations of high-density genotyped Fleckvieh cows were used to assess cross-validation accuracy of genomic predictions with additive and dominance models. The potential use of dominance variance in planned matings was also investigated.

**Results:**

Variance components of nine milk production and conformation traits were estimated with additive and dominance models using yield deviations of 1996 Fleckvieh cows and ranged from 3.3% to 50.5% of the total genetic variance. REML and Gibbs sampling estimates showed good concordance. Although standard errors of estimates of dominance variance were rather large, estimates of dominance variance for milk, fat and protein yields, somatic cell score and milkability were significantly different from 0. Cross-validation accuracy of predicted breeding values was higher with genomic models than with the pedigree model. Inclusion of dominance effects did not increase the accuracy of the predicted breeding and total genetic values. Additive and dominance SNP effects for milk yield and protein yield were estimated with a BLUP (best linear unbiased prediction) model and used to calculate expectations of breeding values and total genetic values for putative offspring. Selection on total genetic value instead of breeding value would result in a larger expected total genetic superiority in progeny, i.e. 14.8% for milk yield and 27.8% for protein yield and reduce the expected additive genetic gain only by 4.5% for milk yield and 2.6% for protein yield.

**Conclusions:**

Estimated dominance variance was substantial for most of the analyzed traits. Due to small dominance effect relationships between cows, predictions of individual dominance deviations were very inaccurate and including dominance in the model did not improve prediction accuracy in the cross-validation study. Exploitation of dominance variance in assortative matings was promising and did not appear to severely compromise additive genetic gain.

## Background

Dominance arises when the effects of alleles at a locus are not only additive, but interact so that the value of the heterozygous genotypes deviates from the mean of the values of the homozygous genotypes. With *a* and –*a* being the genotypic values of homozygous genotypes A_1_A_1_ and A_2_A_2_, let *d* be the genotypic value of the heterozygous genotype A_1_A_2_[1]. If *d* = 0, there is no dominance action at the locus and the genotypic values at the locus are purely additive. The additive effects of genotypes at a locus are expressed as breeding values, which include part of the dominance effect because animals pass alleles, not genotypes, to their offspring. Breeding values are 2*q*[*a* + *d*(*q*-*p*)] for genotype A_1_A_1_, (*q*-*p*)[*a* + *d*(*q*-*p*)] for genotype A_1_A_2_ and -2*p*[*a* + *d*(*q*-*p*)] for genotype A_2_A_2_, where *p* is the frequency of allele A_1_ in the population and *q* the frequency of allele A_2_. The dominance deviation for a given genotype at the locus is the difference between genotypic value and breeding value, and is equal to -2*q*^2^*d*, 2*pqd* and -2*p*^2^*d* for genotypes A_1_A_1_, A_1_A_2_ and A_2_A_2_, respectively [1].

Until recently, studies on dominance deviations were sparse because without genomic information, the availability of large datasets with sufficient proportions of individuals with non-null dominance effect relationships, such as full-sibs, is essential for accurate estimation of dominance variance. Estimates of dominance variance in dairy cattle that are based on pedigree data range from 7.3% to 49.8% of the total genetic variance for conformation traits [2,3] and from 3.4% to 42.9% for milk production traits [4-6].

At the individual animal level, dominance is hardly used in animal breeding [7], although it contains a relevant part of genetic variation. The reasons are the heavy computational demand of large-scale genetic evaluations for dominance, the relatively low accuracy of resulting estimates of dominance effects, and the complexity of planning and computing the outcome of planned matings [8].

With the availability of SNP genotypes, dominance at a marker locus can be readily determined, dominance effects of markers can be estimated [9,10] and computing the expected outcome of planned matings based on SNP genotypes is straightforward [9]. Furthermore, covariance matrices of genomic dominance effects among individuals can be calculated, similar to matrices of genomic additive relationships, which are widely used in genomic selection, such that dominance effects can be estimated in a GBLUP (genomic best linear unbiased prediction) model [11,12].

In this work, we explored the possibilities of including dominance effects in genomic evaluation and furthermore in planned matings in dairy cattle. We estimated variance components, including dominance variance, in a dataset of genotyped Bavarian Fleckvieh cows, analyzed the predictions of breeding and total genetic values using cross-validation, and predicted total genetic values of specific matings.

## Methods

### Estimation of variance components

First-lactating cows from 145 Bavarian dairy herds (all first-lactating cows of each herd were genotyped), born in 2008 and 2009, were genotyped with the Illumina BovineHD Genotyping BeadChip that includes 777 962 SNPs. SNPs with a call rate lower than 0.9, a minor allele frequency higher than 0.005 and a highly significant deviation (*P* < 10^-5^) from the Hardy-Weinberg equilibrium, and SNPs that were not annotated (UMD3) on the autosomes or on the pseudo-autosomal region of the X-chromosome were excluded from the analysis. A total of 629 028 SNPs remained in the dataset after editing. High-density SNP genotypes and yield deviations (YD) for nine traits (milk yield, fat yield, protein yield, somatic cell score, milkability, stature, udder score, udder depth and feet and legs score) from 1996 Bavarian Fleckvieh cows were available to (a) estimate variance components, including dominance variance and (b) perform cross-validation in order to evaluate the predictive ability of a model with dominance effects in comparison to a purely additive model. Both studies were done within a GBLUP framework. YD were calculated based on test-day observations adjusted for non-genetic effects, but not for permanent environmental effects, for each lactation and interpolated by the method of best prediction [13,14]. A weighted mean was calculated across lactation YD of a cow in order to obtain one multi-lactation YD per cow. The effective number of own performances (EOP) [15] was provided as a weight for the multi-lactation YD. For conformation traits, a permanent environmental effect was not modeled because repeated measurements are not available for cows.

Additive genetic ($\sigma_{A}^{2}$) and residual ($\sigma_{E}^{2}$) variance components were estimated with models MA and MG.

$$\begin{array}{l} \mathrm{MA}:y=µ+\mathrm{Zu}+e\\ \mathrm{MG}:y=µ+\mathrm{Zu}+e, \end{array}$$

where **y** is a vector of multi-lactation YD, *μ* is the overall mean, **Z** is a design matrix relating YD to breeding values, **u** is a vector of breeding values of cows, and **e** is a vector of residuals. Covariance matrices of additive effects were $V(u)A\sigma_{A}^{2}$ in model MA and $V(u)=G\sigma_{A}^{2}$ in model MG, where **A** is the numerator relationship matrix and **G** is the genomic relationship matrix. The genomic relationship matrix G^*^ was calculated based on the approach of VanRaden [16] using PREGSF90 [17]:

G*=WaWa'2∑k=1mpkqk,

where matrix **W**_*a*_ has dimensions of the number of individuals (*n*) by the number of loci (*m*), with elements that are equal to 2-2*p*_*k*_ and -2*p*_*k*_ for opposite homozygous and 1-2*p*_*k*_ for heterozygous genotypes, *p*_*k*_ is the minor allele frequency of locus *k*, and *q*_*k*_ =1-*p*_*k*_. Matrix G^*^ was scaled so that the means of diagonals and off-diagonals are the same as in **A**[18,19] and then combined with **A** to **G** = 0.95 G^*^ + 0.05 **A** in order to improve numerical stability. The variance matrix of residual effects was $V(e)\;=\;F\sigma_{E}^{2}$ for both models, where **F** is a diagonal matrix with reciprocals of the EOP as weights. Extending model MG with dominance effects leads to model MGD:

MGD:y=µ+Zu+Zv+e,

where **v** is a vector of dominance deviations of cows. V(**u**) and V(**e**) are defined as in model MG. The covariance matrix of dominance effects is $V(v)=D\sigma_{D}^{2},$ where **D** is the genomic dominance relationship matrix and $\sigma_{D}^{2}$ is the dominance variance. Matrix D^*^ was calculated as:

D*=WdWd'4∑k=1mpk2qk2,

where **W**_***d***_ has dimensions of the number of individuals (*n*) by the number of loci (*m*), with elements that are equal to $-2q_{k}^{2}$ for genotype A_1_A_1_, 2*p*_*k*_*q*_*k*_ for genotype A_1_A_2_, and $-2p_{k}^{2}$ for genotype A_2_A_2_. Matrix D^*^ was then combined with the identity matrix **I** as **D** = 0.95 D^*^ + 0.05 **I** to improve numerical stability.

Estimation of variance components was performed with REMLF90 [20]. Goodness of fit of the respective models to the data was measured by the likelihood. The superiority of model MGD over model MG was tested by a likelihood ratio test, which was calculated as -2ln(likelihood for MG) + 2ln(likelihood for MGD). The likelihood ratio follows a mixture of χ^2^-distributions with 0 and 1 degree of freedom [21]. In addition, variance components of model MGD were estimated by Gibbs sampling using the GIBBS1F90 software [20] in order to compare them with REML results and to calculate standard errors of the estimates. A total of 200 000 iterations of the sampler were run, with the first 20 000 iterations discarded as burn-in samples and every 50^th^ sample included in the posterior analysis. Convergence to the final distribution was checked with the Geweke diagnostics [22] of the R package coda [23,24].

Additive and dominance variance components at the marker level ($\sigma_{a}^{2}$ and $\sigma_{d}^{2}$) were also estimated with the GS3 software [25] in a Markov chain Monte Carlo algorithm, using a model at the marker level (referred to as the MGD-SNP model hereinafter), in contrast to the previous animal level models:

y=1μ+Ta+Xd+e,

where **a** and **d** are vectors of additive and dominant effects of the SNPs, and **T** and **X** are incidence matrices coded as {-1, 0, 1} and {0, 1, 0} for the three possible genotypes. The assumed variance-covariance structure was $V(a)=I\sigma_{a}^{2}$ and $V(d)=I\sigma_{d}^{2}$. From the resulting estimates, additive and dominance variance components on the animal level were calculated as:

$$\begin{array}{l} \sigma_{A}^{2}=\;\sum_{k\;=\;1}^{m}\left(2p_{k}q_{k}\right)\sigma_{a}^{2}\;\\ +\;\sum_{k\;=\;1}^{m}\left[2p_{k}q_{k}{\left(q_{k}-p_{k}\right)}^{2}\right]\sigma_{d}^{2} \end{array}$$

and $\sigma_{D}^{2}=\;\sum_{k\;=\;1}^{m}\left(4p_{k}^{2}q_{k}^{2}\right)\sigma_{d}^{2}$[12].

A total of 300 000 iterations of Gibbs sampling were performed for each trait. The first 20 000 iterations were discarded as burn-in samples and from the remaining 280 000 every 50^th^ sample was considered for analysis of the posterior distribution.

### Prediction of breeding values and total genetic values – cross-validation

Genotyped cows with YD for the respective traits were randomly divided in ten groups in order to perform cross-validation analysis. Typically, splitting at random implies that some validation animals have descendants in the training dataset, which means that the cross-validation is based on descendants, a case of no interest in reality and which will inflate accuracies [26]. In our dataset, genotyped cows were from a single generation. Therefore, a predicted cow could not have daughters (but, e.g., half- or full-sibs) in the training dataset – hence limiting upward bias in the estimation caused by progeny of validation animals in the training data. In this setting, the cross-validation accuracy measures the accuracy to predict contemporary cows including half- and full-sibs of training cows. Each group served once as validation group and the calibration group consisted of the other nine groups. Breeding values and total genetic values for the validation group were predicted based on models MA, MG, and MGD with their respective variance components estimated with REMLF90. The correlation between predicted breeding values and YD in the validation group [$r\left(\mathrm{YD},\hat{u}\right)$] was calculated, as well as the regression of YD on predicted breeding values [$b\left(\mathrm{YD},\hat{u}\right)$]. For model MGD, the correlation between predicted total genetic values and YD [$r\left(\mathrm{YD},\hat{g}\right)$] and the regression of YD on predicted total genetic values [$b\left(\mathrm{YD},\hat{g}\right)$] were also calculated. These measures were averaged over the ten validation groups.

### Prediction of total genetic values of matings

Genotype probabilities and expectations of purely additive breeding values (*u*) and total genetic values (*g*), that include dominance deviations, were calculated for the offspring of all possible matings between 1996 cows and 50 bulls for milk yield and protein yield. The bulls were genotyped and selected for the respective trait on their conventional breeding value after progeny test (including the records of 1996 genotyped cows) from the German-Austrian genetic evaluation. SNP effects *a* and *d* were estimated in a BLUP model (BLUP-SNP; equal to model MGD-SNP but with variance components known) using GS3. Variance components $\sigma_{a}^{2}$ and $\sigma_{d}^{2}$ were fixed to values calculated from REMLF90 variance components $\sigma_{A}^{2}$ and $\sigma_{D}^{2}$ (model MGD):

$$\sigma_{d}^{2}=\frac{\sigma_{D}^{2}}{\sum \left(2^{2}p_{k}^{2}q_{k}^{2}\right)};\;\sigma_{a}^{2}\;=\;\frac{\sigma_{A}^{2}-\sum \left[2p_{k}q_{k}{\left(q_{k}-p_{k}\right)}^{2}\right]\sigma_{d}^{2}}{\sum \left(2p_{k}q_{k}\right)}.$$

The total genetic value *g*_*ij*_ of progeny from a mating between bull *i* and cow *j* was predicted as in Toro and Varona [9]:

$${\hat{g}}_{\mathrm{ij}}=\;\sum_{k}\left[Pr_{\mathrm{ijk}}(\mathrm{AA}){\hat{a}}_{k}\;+\;Pr_{\mathrm{ijk}}(\mathrm{Aa}){\hat{d}}_{k}-Pr_{\mathrm{ijk}}(\mathrm{aa}){\hat{a}}_{k}\right],$$

where *Pr*_*ijk*_() is the probability of the corresponding genotype at locus *k*. Analogously, the breeding value *u*_*ij*_ of progeny from a mating between bull *i* and cow *j* was predicted as:

$$\begin{array}{l} {\hat{u}}_{\mathrm{ij}}=\;\sum_{k}[Pr_{\mathrm{ijk}}(\mathrm{AA})\left(2-2p_{k}\right){\hat{\alpha }}_{k}\;\\ +\;Pr_{\mathrm{ijk}}(\mathrm{Aa})\left(1-2p_{k}\right){\hat{\alpha }}_{k}+Pr_{\mathrm{ijk}}(\mathrm{aa})\left(-2p_{k}\right){\hat{\alpha }}_{k}], \end{array}$$

where ${\hat{\alpha }}_{k}\;=\;{\hat{a}}_{k}\;+\;{\hat{d}}_{k}\left(q_{k}\;-\;p_{k}\right)$_._

Matings can be selected on $\hat{u}$ to maximize additive genetic gain or on $\hat{g}$ to maximize total genetic superiority. The latter maximizes the productive performance of the offspring, which might be a farmer’s interest. However, $\hat{g}$ can be maximized only for the next generation because gain in the dominance part of $\hat{g}$ cannot be accumulated in subsequent generations. In our example, additive gain is assured by pre-selection of bulls on their conventional breeding value. Selection on $\hat{u}$ leads to maximum additive gain, which can be accumulated in subsequent generations, and thus optimizes cumulative multi-generational genetic gain. A desirable objective might be to maximize $\hat{g}$ of matings and at the same time to keep the expected $\hat{u}$ of the offspring as high as possible.

In order to compare the results of these two possible selection strategies, $\hat{g}$ and $\hat{u}$ of all possible matings between the 1996 cows and 50 bulls were calculated for milk and protein yields. For each cow, the top mating was selected with respect to $\hat{g}$ or $\hat{u}$, with the restriction that a single bull was not mated to more than 200 cows. The expected additive genetic gains and total genetic superiorities with selection on $\hat{u}$ or $\hat{g}$ were calculated as the difference between the mean $\hat{u}$ or $\hat{g}$ of selected matings and the mean $\hat{u}$ or $\hat{g}$ of all possible matings.

## Results

### Estimation of variance components

**Figure 1:**
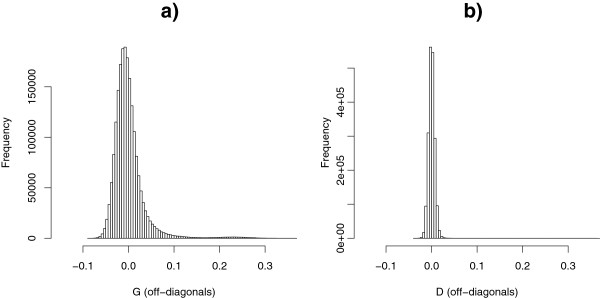
Histograms of off-diagonal elements of relationship matrices G (unscaled) (a) and D (b).

Figure 1 shows the histograms of off-diagonal elements of the additive and dominance genomic relationship matrices. Means of off-diagonals of **G** (before scaling) and **D** were equal to 0, which implies that the population was in Hardy-Weinberg equilibrium. The standard deviation of off-diagonals of **G** was equal to 0.036, which is five times larger than the standard deviation of off-diagonals of **D**, i.e. 0.007. The proportion of off-diagonals that were smaller than -0.05 or larger than 0.05 was 6.27% for **G** but only 0.02% for **D**. Therefore, matrix **D** was less informative than **G**.

Estimated variance components for model MGD are in Table 1. Dominance variance (expressed as a percentage of total genetic variance) for milk production traits ranged from 28.1% for fat yield to 40.9% for protein yield. For somatic cell score and milkability, dominance variance was estimated at 39.0 and 50.5% of the genetic variance. Estimates of dominance variance for conformation traits were quite small, except for udder depth, ranging from 3.3% for stature to 15.3% for feet and legs score. For udder depth, dominance variance was estimated at 23.8% of the genetic variance. For comparison, additive variances estimated with models MA and MG are also in Table 1. With the exception of milkability, the estimates of additive variance from model MG were consistent with additive variance estimates from the dominance model. Estimates of additive variance obtained with the pedigree model MA differed to some extent from those obtained with the genomic models. Estimates of variance components obtained using Gibbs sampling with model MGD and with an equivalent MGD-SNP model are in Table 2 and were similar to REML estimates with model MGD. Geweke statistics [22] showed convergence for model MGD but for the MGD-SNP model, the Gibbs chains did not converge even after 300 000 iterations. However, the means of the Gibbs chains for the MGD-SNP model were similar to those for the MGD model. For stature, udder score and feet and legs score, the estimated dominance variance was clearly larger with both Gibbs sampling analyses than with REML estimation because of a skewed posterior distribution of the Gibbs samples. Estimates of the ratio between dominance and total genetic variance had standard errors around 0.10, which is fairly good for such a small dataset.

**Table 1:** Estimates of additive and dominance variance components obtained using REMLF90 for models MA, MG and MGD

**Trait**	**MA**	**MG**	**MGD**			
	$\sigma_{A}^{2}$	$\sigma_{A}^{2}$	$\sigma_{A}^{2}$	$\sigma_{D}^{2}$	$\sigma_{E}^{2}$	$\frac{\sigma_{D}^{2}}{\sigma_{A}^{2}+\sigma_{D}^{2}}$
Milk yield	261500	214200	208900	92640	164700	0.308
Fat yield	279	274	267	104	198	0.281
Protein yield	213	175	166	115	154	0.409
Somatic cell score	0.230	0.264	0.256	0.261	0.555	0.505
Milkability	0.0193	0.0216	0.0122	0.0076	0.0029	0.390
Stature	3.41	5.73	5.80	0.20	6.51	0.033
Udder score	1.85	2.00	1.99	0.27	9.29	0.118
Udder depth	0.313	0.380	0.380	0.119	0.517	0.238
Feet and legs score	1.32	1.20	1.19	0.21	9.89	0.153

**Table 2:** Estimates of additive and dominance variance components from Gibbs sampling for models MGD and MGD-SNP

**Trait**	**MGD**				**MGD-SNP**			
	$\sigma_{A}^{2}$	$\sigma_{D}^{2}$	$\sigma_{E}^{2}$	$\frac{\sigma_{D}^{2}}{\sigma_{A}^{2}+\sigma_{D}^{2}}$	$\sigma_{A}^{2}$	$\sigma_{D}^{2}$	$\sigma_{E}^{2}$	$\frac{\sigma_{D}^{2}}{\sigma_{A}^{2}+\sigma_{D}^{2}}$
Milk yield	211124 ± 28668	98430 ± 45503	161657 ± 33376	0.306 ± 0.108	202367 ± 23929	115345 ± 32156	152862 ± 27651	0.358 ± 0.067
Fat yield	270.2 ± 36.9	112.5 ± 53.3	193.5 ± 36.9	0.283 ± 0.105	261.9 ± 31.0	119.1 ± 34.4	192.4 ± 29.6	0.308 ± 0.064
Protein yield	168.2 ± 26.3	117.8 ± 43.7	152.7 ± 28.9	0.401 ± 0.105	166.2 ± 23.0	105.6 ± 30.5	160.9 ± 25.0	0.383 ± 0.072
Somatic cell score	0.268 ± 0.067	0.261 ± 0.121	0.554 ± 0.096	0.471 ± 0.155	0.220 ± 0.070	0.130 ± 0.068	0.680 ± 0.088	0.352 ± 0.101
Milkability	0.01228 ± 0.00188	0.00735 ± 0.00169	0.00315 ± 0.00102	0.375 ± 0.082	0.0116 ± 0.00119	0.00724 ± 0.00149	0.00397 ± 0.00123	0.382 ± 0.061
Stature	5.874 ± 0.869	0.616 ± 0.493	6.119 ± 0.802	0.091 ± 0.065	5.754 ± 0.749	1.325 ± 0.497	5.517 ± 0.809	0.184 ± 0.058
Udder score	2.016 ± 0.521	1.089 ± 0.787	8.527 ± 0.921	0.322 ± 0.161	2.010 ± 0.498	1.134 ± 0.452	8.466 ± 0.819	0.352 ± 0.070
Udder depth	0.3852 ± 0.0608	0.1656 ± 0.0952	0.4730 ± 0.1024	0.285 ± 0.120	0.387 ± 0.055	0.181 ± 0.059	0.457 ± 0.082	0.312 ± 0.067
Feet and legs score	1.212 ± 0.451	0.947 ± 0.676	9.209 ± 0.831	0.407 ± 0.192	1.320 ± 0.382	0.936 ± 0.370	9.118 ± 0.700	0.408 ± 0.070

The results are given as estimate ± standard error.

For all traits, model MG, which exploited genomic information, fitted the data better than model MA, which included pedigree information only. The superiority of model MGD, which included a dominance effect, compared to model MG was significant for milk yield, fat yield, protein yield, somatic cell score and milkability, based on the likelihood ratio test. Likelihood measures and statistics of the likelihood ratio test between models MG and MGD are in Table 3. The likelihood ratio test statistics were asymptotically χ^2^-distributed [27]. The χ^2^-distribution function can take only non-negative values because it is defined as a sum of squared values. For two traits (stature and udder score), the likelihood ratio test statistics were negative (but very close to 0), which was due to numerical rounding or not finding the mode of the likelihood exactly.

**Table 3:** **Goodness of fit of models MA, MG, and MGD and likelihood ratio test (χ**^**2**^**-value and P-value) between models MG and MGD**

	**-2 log likelihood**			**Likelihood ratio test**	
	**MA**	**MG**	**MGD**	**χ**^**2**^**-value**	**P-value**
Milk yield	31531.5	31488.1	31484.3	3.8	0.026
Fat yield	18363.1	18299.5	18295.9	3.6	0.029
Protein yield	17852.5	17824.6	17817.6	7.0	0.004
Somatic cell score	6072.9	6055.0	6050.6	4.4	0.018
Milkability	-1243.5	-1297.6	-1323.9	26.3	1.46*10^-7^
Stature	9979.0	9907.9	9908.4	-0.5	1.000
Udder score	9916.5	9902.4	9902.5	-0.1	1.000
Udder depth	5287.9	5239.7	5238.5	1.2	0.137
Feet and legs score	9884.9	9880.9	9880.9	0.0	1.000

The measures of goodness of fit (-2 log likelihood) for models MA, MG and MGD are reported as well as the likelihood ratio test statistics ($\mathrm{\chi ²}-\text{value}\;=\;-2\ln\frac{\text{likelihood}\;\text{for}\;\mathrm{MG}}{\text{likelihood}\;\text{for}\;\mathrm{MGD}}$) between models MG and MGD and the corresponding P-values.

### Prediction of breeding values and total genetic values – cross-validation

Mean accuracies of predicted breeding values [$r\left(\mathrm{YD},\hat{u}\right)$] and slopes of the regression of YD on predicted breeding values [$b\left(\mathrm{YD},\hat{u}\right)$] are in Table 4. For model MA, $r\left(\mathrm{YD},\hat{u}\right)$ ranged from 0.102 for somatic cell score to 0.228 for fat yield, with an average of 0.165. Replacing pedigree with genomic relationships increased $r\left(\mathrm{YD},\hat{u}\right)$ to between 0.108 (feet and legs) and 0.327 (milkability), with an average of 0.242. $r\left(\mathrm{YD},\hat{u}\right)$ did not change when dominance effects were added to the model. Average standard errors of $r\left(\mathrm{YD},\hat{u}\right)$ were equal to 0.024, 0.021 and 0.021 in models MA, MG and MGD, respectively. $r\left(\mathrm{YD},\hat{u}\right)$ with the dominance model ranged from 0.109 for feet and legs score to 0.325 for fat yield. The difference between $r\left(\mathrm{YD},\hat{g}\right)$ and $r\left(\mathrm{YD},\hat{u}\right)$ in model MGD ranged from -0.004 for protein yield to 0.003 for udder score. The standard errors of $r\left(\mathrm{YD},\hat{g}\right)$ were similar to those for $r\left(\mathrm{YD},\hat{u}\right)$, with a mean of 0.021.

**Table 4:** Accuracies and regression coefficients of predicted breeding values and total genetic values for models MA, MG, and MGD

**Trait**	${r\left(\mathrm{YD},\hat{u}\right)}^{1}$			${r\left(\mathrm{YD},\hat{g}\right)}^{2}$	${b\left(\mathrm{YD},\hat{u}\right)}^{3}$			${b\left(\mathrm{YD},\hat{g}\right)}^{4}$
	**MA**	**MG**	**MGD**	**MGD**	**MA**	**MG**	**MGD**	**MGD**
Milk yield	0.221 ± 0.029	0.277 ± 0.030	0.278 ± 0.031	0.275 ± 0.032	0.925 ± 0.109	0.955 ± 0.099	0.967 ± 0.101	0.950 ± 0.104
Fat yield	0.228 ± 0.018	0.325 ± 0.020	0.325 ± 0.019	0.325 ± 0.019	1.031 ± 0.085	1.068 ± 0.078	1.085 ± 0.079	1.072 ± 0.075
Protein yield	0.202 ± 0.031	0.236 ± 0.016	0.238 ± 0.016	0.234 ± 0.016	0.958 ± 0.148	0.889 ± 0.070	0.924 ± 0.072	0.889 ± 0.069
Somatic cell score	0.102 ± 0.018	0.169 ± 0.020	0.169 ± 0.019	0.168 ± 0.015	0.866 ± 0.165	1.007 ± 0.131	1.031 ± 0.133	0.973 ± 0.107
Milkability	0.133 ± 0.042	0.327 ± 0.025	0.324 ± 0.028	0.322 ± 0.027	0.563 ± 0.182	0.744 ± 0.057	1.053 ± 0.099	1.004 ± 0.087
Stature	0.180 ± 0.017	0.308 ± 0.014	0.308 ± 0.014	0.308 ± 0.014	1.082 ± 0.117	1.030 ± 0.059	1.023 ± 0.059	1.021 ± 0.059
Udder score	0.121 ± 0.017	0.159 ± 0.022	0.159 ± 0.022	0.158 ± 0.022	1.023 ± 0.144	1.004 ± 0.142	1.007 ± 0.142	1.002 ± 0.146
Udder depth	0.192 ± 0.017	0.269 ± 0.020	0.269 ± 0.020	0.272 ± 0.020	1.031 ± 0.119	0.988 ± 0.095	0.991 ± 0.094	0.988 ± 0.095
Feet and legs score	0.106 ± 0.026	0.108 ± 0.023	0.108 ± 0.023	0.109 ± 0.024	1.201 ± 0.290	1.055 ± 0.221	1.063 ± 0.223	1.060 ± 0.225

The results are given as mean ± standard error.^1^$r\left(\mathrm{YD},\hat{u}\right)$ = accuracy of predicted breeding values (cross-validation correlation between YD and predicted breeding values).^2^$r\left(\mathrm{YD},\hat{g}\right)$ = accuracy of predicted total genetic values (cross-validation correlation between YD and predicted total genetic values).^3^$b\left(\mathrm{YD},\hat{u}\right)$ = regression coefficient of YD on predicted breeding values.^4^$b\left(\mathrm{YD},\hat{g}\right)$ = regression coefficient of YD on predicted total genetic values.

For models MA and MG, $b\left(\mathrm{YD},\hat{u}\right)$ ranged from 0.563 (milkability) to 1.201 (feet and legs score) and from 0.744 (milkability) to 1.068 (fat yield), respectively, with means of 0.964 and 0.971. $b\left(\mathrm{YD},\hat{u}\right)$ for model MGD ranged from 0.924 (protein yield) to 1.085 (fat yield), with a mean of 1.016. The standard errors of $b\left(\mathrm{YD},\hat{u}\right)$ were rather large, with means of 0.151, 0.106 and 0.111 for models MA, MG and MGD, respectively. The slope of the regression of YD on predicted total genetic values ranged from 0.889 (protein yield) to 1.060 (feet and legs score), with a mean of 0.995 and was slightly smaller than $b\left(\mathrm{YD},\hat{u}\right)$ for most traits for the same model. The fact that slopes were generally not significantly different from 1 suggests that predictions were essentially unbiased, except for milkability.

### Prediction of total genetic values of matings

For milk yield, 16 bulls were chosen as mating partners when matings were selected on $\hat{g}$. The restriction of at most 200 cows per bull was reached for seven bulls. The remaining nine bulls were mated to 197, 147, 139, 86, 19, 4, 2, 1 and 1 cows. When matings were selected on $\hat{u}$, nine bulls were mated to the maximum number of 200 cows and two other bulls to 176 and 20 cows, respectively. For protein yield, 24 bulls were chosen as mating partners when matings were selected on $\hat{g}$. The restriction of 200 cows per bull was reached for seven bulls. The remaining 17 bulls were mated to 134, 115, 114, 63, 62, 29, 26, 21, 8, 7, 4, 3, 3, 3, 2, 1 and 1 cows. When matings were selected on $\hat{u}$, eight bulls were mated to the maximum number of 200 cows and the four other bulls to 190, 157, 33 and 16 cows.

Expected total genetic superiorities and additive genetic gains obtained with the selected matings are in Table 5, both in absolute numbers and relative to the standard deviations (SD) of $\hat{u}$ and $\hat{g}$. When matings were selected on $\hat{g}$ for milk yield, the expected total genetic superiority was estimated to be equal to 165.2 kg, which is equivalent to 1.01 SD of $\hat{g}$. The expected total genetic superiority was reduced to 143.8 kg (0.88 SD) when matings were selected on $\hat{u}$. The expected additive genetic gain was less sensitive to the selection criterion applied since it was only slightly reduced when selection was done on $\hat{g}$ (137.7 kg; 0.85 SD) instead of on $\hat{u}$ (143.8 kg; 0.89 SD). The results were similar for protein yield. With selection on $\hat{g}$, the expected additive genetic gain was slightly smaller (0.74 vs. 0.76 SD) but the expected total genetic superiority was clearly larger (1.01 vs. 0.79 SD) compared to selection on $\hat{u}$.

**Table 5:** **Expected total genetic superiority (ΔG) and additive genetic gain (ΔU) with selection on total genetic value (**$\hat{g}$**) or breeding value (**$\hat{u}$**)**

	**ΔG**		**ΔU**	
	**absolute (kg)**	**relative to SD**	**absolute (kg)**	**relative to SD**
Milk yield				
Selection on $\hat{g}$	165.2	1.01	137.7	0.85
Selection on $\hat{u}$	143.8	0.88	143.8	0.89
Protein yield				
Selection on $\hat{g}$	4.15	1.01	3.09	0.74
Selection on $\hat{u}$	3.24	0.79	3.16	0.76

Expected total genetic superiority (ΔG) and expected additive genetic gain (ΔU) for the alternative selection criteria total genetic value ($\hat{g}$) and breeding value ($\hat{u}$) in absolute value (kg) and relative to the standard deviations (SD) of $\hat{g}$ and $\hat{u}$ of all possible matings; the maximum number of matings per bull was restricted to 200.

## Discussion

This study analyzed the importance of dominance variation for several milk production and conformation traits in the Fleckvieh breed using the GBLUP methodology. Additive and dominance genomic relationship matrices were calculated similar to Su et al. [11], except that standard quantitative genetic approaches were used, with the dominance variance at locus *k* defined as (2*p*_*k*_*q*_*k*_*d*)^2^[1,12]. This resulted in the reported estimates of dominance variance to be compatible with pedigree-based estimates.

Independence between **u** and **v** is the classical treatment [1] and it is convenient because it allows orthogonality of the estimates and thus an easy translation into variances and covariances of **u** and **v**. However, this independence is contradictory with the phenomena of inbreeding depression and hybrid vigor; presence of inbreeding depression indicates that dominance is directional, e.g. [28]. Wellmann and Bennewitz [10,29] reviewed biological information on milk yield and productive life in Holstein cattle to suggest *a priori* dependencies between *a* and *d* (which would result in dependencies between **u** and **v**) and Bayesian regression models that could accommodate those dependencies. The treatment of dependencies between breeding values and dominance deviations is rather complex and the computational requirements are large, thus, we did not consider this method although it should be a field of further research.

Estimates of dominance variance varied from 3.3 to 50.5% of total genetic variance for the analyzed traits. Estimated dominance variance (as a percentage of total genetic variance) was greater for milk production traits than for conformation traits. These results agree with those of Misztal et al. [7], who found larger dominance variance for production than for conformation traits. Moreover, Misztal et al. [3] reported estimates of dominance variance in US Holstein cattle for 14 conformation traits that ranged from 7.3 (rump angle) to 22.3% (strength) of the total genetic variance. This is comparable to the estimates of dominance variance for the conformation traits analyzed in this study. In the literature, reported estimates of dominance variance for milk production traits of Holstein cattle vary considerably ranging from 1.4 to 42.9% of the total genetic variance [4-7], which are within the same range but smaller than those found in our study. Two reasons may explain the relatively large estimates of dominance variance for milk production traits obtained in our study compared to values reported in the literature: (1) Fleckvieh cattle are genetically more diverse than Holstein cattle, as reflected by the considerably larger effective population size of the Fleckvieh breed [30], which is expected to result in more heterozygosity and in QTL alleles with more intermediate frequencies; (2) all estimates of dominance variance available in the literature were obtained using relationship matrices based on pedigree data; the use of genomic information is expected to improve estimates of dominance effect relationships and reduce potential confounding with additive effects and residuals which is likely to result in different estimates.

Although moderate changes in estimates of additive variance were observed between pedigree and genomic models, estimates of additive variance were consistent for genomic additive and dominance models, except for milkability. Su et al. [11] reported a small difference in estimates of additive variance between additive and dominance models. However, the additive and dominance variances reported in Su et al. [11] result from an alternative partitioning of genetic variance and are thus not directly comparable to the classical partitioning of genetic variance [12]. In studies based on pedigree information, estimates of additive variance have been similar between additive and dominance models [5,6,31].

Both Gibbs sampling with model MGD and at the marker level with the MGD-SNP model resulted in estimated variance components that were comparable with REML estimates for most traits. The relative standard error (calculated as standard error divided by the estimate) of dominance variance was on average 2.7 times larger than the relative standard error of the estimated additive variance, which is expected based on the properties of **G** and **D**. However, in other studies the ratio between relative standard errors of dominance and additive variances was even larger, i.e. 4.1 in Misztal [32] and 4.5 in Su et al. [11]. In order to estimate dominance variance more accurately, more dominance-specific information is needed. This could be achieved, e.g., by increasing the number of full-sibs in the dataset. The present dataset contained 3% full-sibs.

Despite the large estimates of dominance variance for most analyzed traits (significantly larger than 0 for five traits), prediction accuracy of breeding values and total genetic values did not change when dominance effects were included in the model. Estimates of additive variance did not differ much between models MG and MGD, which means that additive variance is already captured quite accurately in the additive model. Thus, additive effects are relatively well predicted, whether the dominance effect is modeled or not. The accuracy of predictions of total genetic values in cross-validation was not higher with the dominance than with the additive model because the proportion of full-sibs and dominance effect relationship coefficients between the training and validation datasets were small. Thus, little information was transferred from the reference to the validation group in cross-validation for prediction of dominance effects. Su et al. [11], who analyzed non-additive effects for average daily gain with a dataset of 1911 purebred pigs, observed that the estimates of the additive variance with the additive and dominance models remained fairly constant and that gains in accuracies of predicted breeding values and predicted total genetic values reached only 0.004 and 0.011 with the dominance model. The proportion of full-sibs in the pig dataset was not reported in Su et al. [11] but is expected to be substantially larger than in our cow dataset, which might be the reason for the gain in accuracy of predicted total genetic values with inclusion of dominance in the model. Based on a simulation study, Varona et al. [33] observed that relevant changes in breeding values when switching from an additive to a dominance model were obtained only for animals that had full-sibs or full-sib progeny and little other information. A cow dataset with a larger proportion of full-sibs would contain more information in order to accurately estimate dominance effects but in practice such data is not available. Analysis of full-sib progeny from elite animals, which generally are available, would not be representative for the whole population.

The regression coefficient of YD on predicted breeding values was generally close to 1, with a few exceptions. With the dominance model, this regression coefficient was slightly closer to 1 for most traits but differences were small, which is similar to the data reported by Su et al. [11], i.e. 0.927 and 0.983 with the additive and dominance models, respectively. In our study, the regression coefficient of YD on predicted total genetic values for model MGD was slightly smaller than the regression on predicted breeding values, which agrees with Su et al. [11], but it remained close to the expectation, which means that predictions were unbiased. In general, bias can originate from preferential treatment, unrecognized pre-selection of validation animals, or inappropriate modeling of predictions (i.e. using incorrect variance components).

The results show that selection of matings on $\hat{g}$ instead of $\hat{u}$ led to 14.8% (milk yield) and 27.8% (protein yield) greater expected total genetic superiorities and maximized expected productive performance of the offspring. Although the accuracy of estimates of total genetic values was not greater than that of estimates of breeding values, as indicated by the cross-validation results (Table 4), expected total genetic superiority was not impaired by this result because predicted genetic values are best linear unbiased predictions and therefore unbiased expectations [34]. Toro and Varona [9] reported that expected total genetic superiority with optimized mate allocation was 16% greater than with selection on the breeding value only, for a trait with additive and dominance variances amounting to 40 and 10% of the phenotypic variance. Expected additive genetic gain was reduced by only 4.5% for milk yield and by 2.6% for protein yield with selection of matings on $\hat{g}$ instead of $\hat{u}$. Thus, optimization of $\hat{g}$ of the offspring appears to be feasible without a great loss in $\hat{u}$. Our considerations of optimized matings are limited to the first generation offspring. Toro and Varona [9] found that response from assortative mating was only realized in the first generation without any additional response in subsequent generations. Thus, optimization of matings with respect to total genetic value has to be applied in each generation, otherwise the dominance-specific advantage is lost. Toro and Varona [9] pre-selected males and females on their estimated breeding values and then optimized the total genetic value of matings between these pre-selected animals. In our example, only bulls were pre-selected on their conventional breeding value and the optimal bull was determined for each cow based on the expected total genetic value of the offspring. However, the potential of assortative mating to exploit dominance variance optimally by combining mates that are expected to produce offspring with large total genetic values is limited even for these two traits with sizeable dominance variation. This can be caused either by cancellation effects across the genome (i.e., it is extremely unlikely to combine all positive dominance effects) or by a reduced accuracy of the dominance deviation of a mating because of uncertainty about the resulting marker genotypes.

## Conclusions

Estimates of genomic variance due to dominance in Fleckvieh cattle ranged from 3 to 50% of the genetic variance and were within the range of published pedigree-based estimates for dairy cattle. The computational complexity and modeling were straightforward. Predictive ability of breeding and total genetic values by cross-validation was not improved when dominance effects were included in the prediction model, probably because of the limited size of the dataset and the small proportion of full-sibs. There is potential to exploit dominance variance in planned matings in order to increase total genetic value of the offspring (i.e. future performance) without compromising additive genetic gain. Use of planned matings could also be a way to motivate farmers that are otherwise not interested in using genomic breeding values for breeding schemes.

## Competing interests

The authors declare that they have no competing interests.

## Authors’ contributions

JE performed the analysis and drafted the manuscript. JE, AL and KUG designed the study. AL, ZGV and LV developed methods. CE and RE prepared phenotypic and genotypic data. AL, ZGV, LV, CE, RE and KUG revised the manuscript. All authors read and approved the final manuscript.
